# Low Grade Fibromyxoid Sarcoma in Thigh

**DOI:** 10.4055/cios.2009.1.4.240

**Published:** 2009-11-25

**Authors:** Bong-Jin Lee, Woo-Sung Park, Jong-Mun Jin, Chang-Won Ha, Sang-Hoon Lee

**Affiliations:** Department of Orthopaedic Surgery, Cheju Halla General Hospital, Jeju, Korea.; * Department of Pathology, Cheju Halla General Hospital, Jeju, Korea.

**Keywords:** Low grade fibromyxoid sarcoma, Thigh

## Abstract

A low grade fibromyxoid sarcoma is a rare soft tissue tumor that has a tendency to develop in the deep soft tissue of young adults and the potential for local recurrence or distant metastasis. There have been several case reports and sporadic reports in the literature. However, only 1 case has been reported in Korea but without a follow-up result. We describe a 54-year-old female patient with a low-grade fibromyxoid sarcoma of the thigh that had been growing slowly for 34 years. A marginal resection of this tumor was performed. Currently, the patient is doing well without evidence of local recurrence or distant metastasis at 5 years after surgery.

A low grade fibromyxoid fibrosarcoma is a recently recognized, uncommon soft tissue neoplasm. The condition was reported originally by Evans in 1987, who subsequently described ten additional cases in 1993. Since then, a few sporadic case reports and series have been reported.^1^-7) We describe the MRI and histology findings associated with the clinical results of a 5 year follow-up in a 54-year-old female patient with a low-grade fibromyxoid sarcoma and discuss the differential diagnosis with a review of the relevant literature.

## CASE REPORT

A 54-year-old female complained of a slowly growing mass in her thigh over a 34 year period. She has a two year history of hemodialysis due to chronic renal failure. A physical examination demonstrated a small melon sized mass on the anterior side of the distal thigh. The mass was not tender but rubbery hard and mobile.

**Fig. 1:**
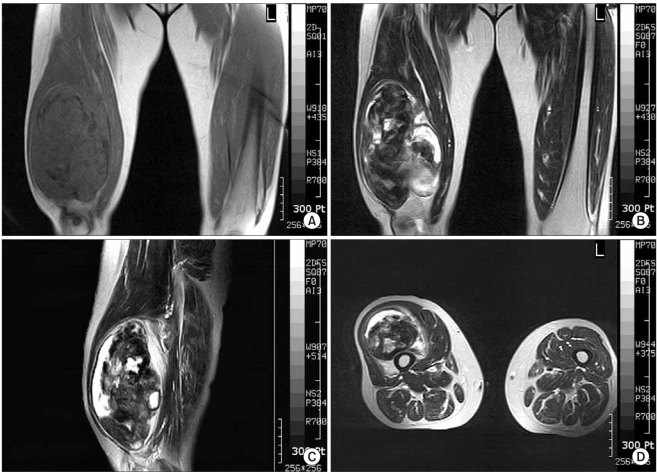
On MRI, the tumor is a 17 × 11 × 9 cm sized mass located under the quadriceps muscle. The tumor matrix is partially calcified and relatively well defined one having irregular low signal intensity in the T1 weighted image (A) and heterogeneous low signal and high signal in the T2 weighted image (B-D).

The plain radiographs revealed a huge soft tissue mass with irregular calcification in the distal thigh. On MRI, the tumor was 17 × 11 × 9 cm in size located under the quadriceps muscle. The tumor matrix was partially calcified and relatively well defined with irregular low signal intensity in the T1 weighted image and heterogeneous low and high signal in the T2 weighted image. There was no abnormal signal of the underlying bony cortex and bone marrow (Fig. 1).

Grossly, the excised tumor was a well circumscribed, lobulated round and firm mass. The cut surface was yellowish-white and fibrous to myxoid without any areas of hemorrhage or necrosis.

**Fig. 2:**
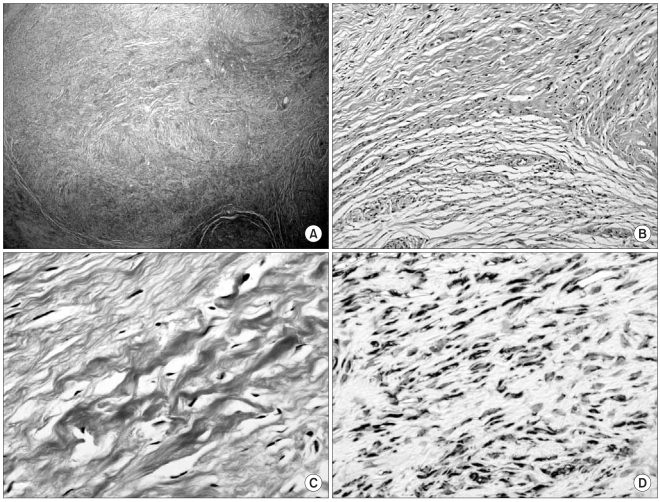
Optical microscopy examination demonstrates a mass with sharp demarcation, nodular growth pattern and intervening hypocellular collagenous stroma (A: Hematoxylin and eosin stain, ×100). The tumor shows a biphasic pattern with fibrous and myxoid areas, minimal nuclear pleomorphism, low to moderate cellularity, and a swirling, whorled growth (B: Hematoxylin and eosin stain, ×200). The background matrix ranges from fibromyxoid to densely fibrous (C: Hematoxylin and eosin stain, ×400). Immunohistochemically, the tumor cells are diffusely and strongly positive for vimentin (D: Immunohistochemical stain for vimentin, ×1400).

The optical microscopy examination demonstrated a mass with sharp demarcation, a nodular growth pattern and intervening hypocellular collagenous stroma (Fig. 2A). The tumor showed a biphasic pattern with fibrous and myxoid areas. The cells were small, bland, regular, fibroblastic spindle cells with minimal nuclear pleomorphism, low to moderate cellularity, and swirling, whorled growth (Fig. 2B). The background matrix ranged from fibromyxoid to densely fibrous (Fig. 2C).

Immunohistochemically, the tumor cells were diffusely and strongly positive for vimentin, but were negative for cytokeratin, smooth muscle actin, S-100 protein and neuron specific enolase. The tumor was identical immunohistochemically to a low grade fibromyxoid sarcoma with strong positivity to vimentin (Fig. 2D).

**Fig. 3:**
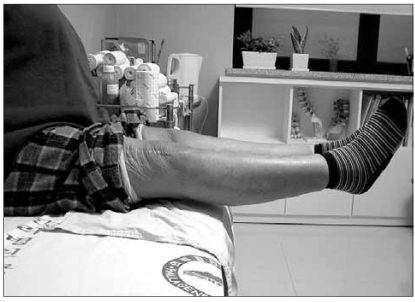
The patient is still alive with no evidence of local recurrence and distant metastasis five years after excision. The range of motion of the knee joint is full and the strength of the quadriceps muscle is normal.

Five years after the excision, the patient is alive with no evidence of local recurrence or distant metastasis. The ranges of knee and hip joint motion were normal and the strength of the quadriceps muscle was good (Fig. 3).

## DISCUSSION

Low grade fibromyxoid sarcoma is a bland soft tissue tumor histologically. The tumor has aggressive behavior in that a complete excision does not necessarily prevent the development of local recurrence or distant metastasis many years later.

Among the 27 cases reviewed, 16 showed local recurrences and 9 had lung metastases.^1^-7) The interval to local recurrence varied from 2 to 13 years (median, 4 years). The interval to the lung metastasis ranged from zero (metastases at presentation) to 45 years (median, 5 years).^4^,5) Moreover, 1 case became dedifferentiated at 30 years after surgery.4) Therefore, it was believed that a minimum 5 year follow-up will be needed to discuss the results of this tumor.

A review of the literature revealed the mean age at the first operation to be 33.2 years (range, 6 to 65 years) with 66.7% being male. Frequent sites were the thigh, shoulder, inguinal area and chest wall (Table 1). The preoperative duration varied from 3 days to 22 years and that of the current case (34 years) was the longest.^1^-7)

The main pathologies to be considered in a differential diagnosis include desmoid fibromatosis, peripheral nerve sheath tumor, myxoid liposarcoma, spindle cell liposarcoma and low grade myxofibrosarcoma.^3^-6)

Desmoid fibromatosis is poorly defined and shows a greater degree of cellularity, prominent fascicular proliferation, and more interstitial collagen fibers. Low grade fibromyxoid sarcoma can be distinguished readily from a nerve sheath tumor by the absence of the S-100 protein or an association with any nerve, and a lesser degree of waviness of the individual nuclei than a nerve sheath tumor.

Low grade fibromyxoid sarcoma lacks lipoblast and arborizing vascular vessels, which are characteristic of myxoid liposarcomas. The absence of adipose elements excludes the possibility of spindle cell liposarcoma.

Low grade myxofibrosarcoma differs from low grade fibromyxoid sarcoma in that it generally occurs in older adults, is always predominantly myxoid and composed of fusiform cells with hyperchromatic atypical nuclei, and does not metastasize.

Low grade fibromyxoid sarcoma is a distinct neoplasm with a tendency to recur or metastasize. Histologically, it is characterized by the presence of bland spindle cells with a mainly whorled pattern of growth, set in alternating areas with a myxoid or fibrous stroma. Immunohistochemical studies allow a discrimination of this entity from other benign and malignant lesions.

We report a patient with of low grade fibromyxoid sarcoma, who was treated with a marginal resection, and had no sign of recurrence or metastasis during a 5 year follow-up. However, close observation will be needed for several decades.

## Figures and Tables

**Table 1:**
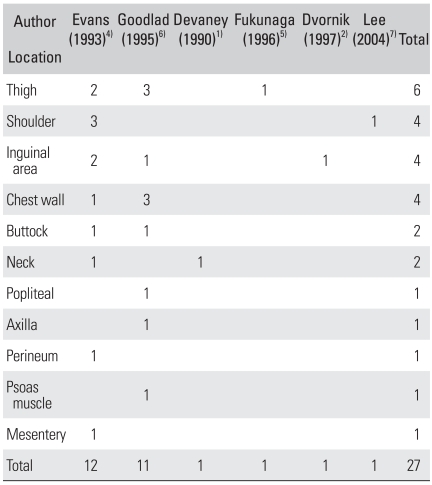
The Location of Low Grade Fibromyxoid Sarcomas
